# Targeting Myocardial Mechanics in Children and Adolescents with Obesity and Non-Elevated Blood Pressure: A Meta-Regression Study

**DOI:** 10.3390/diseases13090301

**Published:** 2025-09-11

**Authors:** Andrea Faggiano, Elisa Gherbesi, Carla Sala, Stefano Carugo, Guido Grassi, Marijana Tadic, Cesare Cuspidi

**Affiliations:** 1 Department of Cardio-Thoracic-Vascular Diseases, Foundation IRCCS Ca’ Granda Ospedale Maggiore Policlinico, 20122 Milan, Italycarla.sala@unimi.it (C.S.);; 2 Department of Clinical Sciences and Community Health, University of Milano, 20122 Milano, Italy; 3 Department of Medicine and Surgery, University of Milano-Bicocca, 20126 Milano, Italy; 4 University Heart Center Ulm, University Ulm, Albert-Einstein Allee 23, 89081 Ulm, Germany

**Keywords:** global longitudinal strain, global circumferential strain, pediatric obesity, systolic function

## Abstract

Background/Objectives: Although global longitudinal strain (GLS) appears more sensitive than the ejection fraction in uncovering subtle left ventricular (LV) dysfunction, evidence of impaired LV mechanics in children/adolescents with obesity, independent of comorbidities, remains limited. The aim of the present study was to provide new information on clinical and echocardiographic correlates associated with LV mechanics in normotensive children/adolescents with obesity and without comorbidities. Methods: The Pub-Med, Ovid MEDLINE, Ovid EMBASE, and Cochrane databases were searched to identify eligible studies from inception up to 31 March 2025. Studies reporting data on LV mechanics (i.e., GLS, global circumferential strain [GCS]) in children/adolescents with obesity were included. Meta-regression analyses between GLS, GCS, and several clinical, laboratory, and echocardiographic parameters were performed using a random-effect model. Results: Twenty-seven studies including 1398 normotensive children/adolescents with obesity (mean age 12.6 ± 1.8 years) were considered. There was a significant inverse relationship between GLS and body mass index (BMI) (coefficient = −0.33 ± 0.11, *p* = 0.003) and fat mass (coefficient = −0.19 ± 0.07, *p* = 0.005); this was not the case for GCS. Notably, both GLS and GCS were unrelated to several clinical/laboratory variables such as blood pressure, metabolic parameters, LV mass, and LV diastolic function indices. Conclusions: Our findings suggest that increasing BMI and fat mass are the only key factors associated with reduced longitudinal myocardial deformation in pediatric obesity. GLS, unlike GCS, can be regarded as an early marker of subclinical organ damage in this setting and should be assessed to optimize cardiovascular prevention strategies in children/adolescents with obesity regardless of hypertension or comorbidities.

## 1. Introduction

Nowadays, obesity and overweight represent one of the main challenges for public health, both in Europe and the rest of the world [1,2,3,4]. The researchers of the Global Burden of Disease Study have highlighted that excess weight has more than doubled in the last thirty years [5]. Globally, overweight and obesity in 2021 have reached the dramatic figure of 2.11 billion adults and 493 million young people, compared to 731 million and 198 million recorded in 1990. According to epidemiological projections for the next few decades, obesity is becoming the more prevalent condition compared to overweight. In particular, between 2022 and 2030, obesity in children and adolescents between the ages of 5 and 14 will surpass overweight, leading to a “global transition to obesity” among young generations. It has been predicted that there will be a 121% increase in obesity among children and adolescents, with the global number of young people with obesity reaching 360 million by 2050.

A large body of evidence based on observational studies has indicated that obesity has deleterious multi-organ effects [6,7,8] and, in particular, represents a powerful risk factor for the development of heart failure (HF) [9,10]. The persistence of overweight/obesity from childhood onwards has been shown to be linked to an increased risk of incident HF, and this association is particularly strong for the preserved ejection fraction phenotype (HFpEF) [11]. The Irbesartan in HF with Preserved Ejection Fraction (I-PRESERVE) trial, including over 70% of participants with BMI > 26.5%, documented that the risk of death or cardiovascular (CV) hospitalization, after adjustment for several confounders, was 27% higher in participants with BMI ≥ 35 kg/m^2^, compared with the reference group [12]. A large meta-analysis comprising 23 observational studies with a pooled population of 647,888 participants revealed that the relative risk for a five-unit increment in BMI was 1.41 (95% CI: 1.34–1.47) for HF incidence and 1.26 (95% CI: 0.85–1.87) for HF mortality [13]. It is noteworthy that this study found an increased risk of HF even in the overweight BMI range.

The pathophysiological mechanisms linking obesity to HF through structural and functional cardiac changes are very complex, with this condition often being associated with a wide array of comorbidities such as hypertension, metabolic syndrome, diabetes mellitus, sleep apnea, and subclinical atherosclerosis and, in terms of other factors, unhealthy dietary patterns, physical inactivity, and unfavorable social and economic status [14,15]. In this scenario, pediatric obesity represents a physio-pathological model of great interest as it allows us to analyze its early impact on cardiac structure and function and, to some extent, independently from the above-mentioned comorbidities that occur progressively with increasing age.

Numerous observational studies have focused on the relationship between obesity in different age groups (from childhood to old age) and echocardiographic phenotypes of subclinical organ damage (mainly represented by left ventricular hypertrophy (LVH) and indices of diastolic dysfunction), suggesting that even in childhood obesity, an increase in the LV mass index (LVMI) may occur in association with a concentric LV geometry, left atrial dilatation, and reduction in LV relaxation and filling [16,17,18]. Conversely, a large amount of evidence on LV systolic function, assessed using the left ventricular ejection fraction (LVEF) metric, supported the view, with some exceptions, that myocardial contractility in children/adolescents with obesity remains substantially preserved [17,18,19,20]. In the last decade, however, the findings documenting normal LV systolic function in pediatric obesity have been challenged by studies on LV mechanics assessed through speckle-tracking echocardiography (STE), which demonstrated subclinical myocardial dysfunction despite preserved ejection fraction [21,22]. In particular, global longitudinal strain (GLS) targeting LV longitudinal deformation as a quantitative assessment of global LV systolic function has been increasingly reported to be more sensitive, reproducible, and less load-dependent than LVEF [23,24].

Findings from STE based studies in children/adolescents with obesity and normal LVEF have consistently shown that longitudinal myocardial deformation is impaired, suggesting that early alterations in LV systolic function, not detectable with conventional echocardiography, may occur very early in this setting [25,26]. GLS primarily reflects the subendocardial longitudinal fibers, which are particularly vulnerable to metabolic and hemodynamic stressors [27,28]. In children with obesity, these alterations appear independent of blood pressure (BP) [20]. By contrast, global circumferential strain (GCS), which reflects mid-wall fibers, can often be preserved, possibly due to compensatory mechanisms that maintain overall pump function in the early stages [29].

Starting from this basis, the present study aimed to investigate through meta-regression analysis the factors associated with alterations of cardiac mechanics in pediatric obesity in order to provide useful indications for potential strategies to prevent/reduce the risk of subclinical cardiac damage, the early onset of which can influence the development of HF in adulthood.

Because arterial hypertension is a major determinant of LV remodeling and systolic dysfunction, we deliberately restricted the analysis to normotensive children and adolescents with obesity. This strategy allowed us to better isolate the independent contribution of adiposity to LV mechanics, minimizing confounding effects.

In this systematic review and meta-regression, we examined children and adolescents with obesity (Population) to assess how adiposity, hemodynamic, metabolic, and echocardiographic factors (Exposures) relate to left ventricular mechanics measured by GLS and GCS (Outcomes).

## 2. Methods

The present study was based on pooled data collected as part of a comprehensive search of available literature on the measurement of cardiac structural and functional parameters by means of conventional echocardiography and STE (i.e., multidirectional LV strain) in children/adolescents with obesity and in healthy subjects of a normal weight.

### 2.1. Methodology of the Primary Analysis

The primary analysis (a systematic review and meta-analysis), aimed to evaluate early alterations in LV mechanics—specifically GLS—in children and adolescents with overweight or obesity and preserved LVEF.

A comprehensive search was performed across PubMed, Ovid MEDLINE, Ovid EMBASE, and the Cochrane Library from inception up to 31 March 2025. Eligible studies included clinical investigations reporting GLS values obtained via 2D or 3D STE in children/adolescents (aged > 3 years) with overweight/obesity and preserved LVEF, compared with healthy normal-weight controls. Only peer-reviewed articles with a minimum dataset of clinical and demographic variables were included. Searches were limited to clinical investigations published in English.

Studies were identified by using MeSH terms and crossing the following search items: “pediatric obesity”, “obesity”, “heart”, “cardiac disease”, “myocardial strain” “left ventricular mechanics”, “global longitudinal strain”, “speckle tracking echocardiography”, “systolic dysfunction”, “left ventricular ejection fraction”. Checks of the reference lists of original papers and pertinent review articles were also searched for additional relevant literature. Data were examined and extracted by three independent investigators (EG, AF, and CC). In case of no agreement on a specific record, the full text of the study was analyzed by all reviewers in order to establish its eligibility according to the inclusion criteria mentioned below. An artificial intelligence tool (Consensus: https://consensus.app/) was used to verify if any studies had been missed by the original search. The main inclusion criteria were (I) English articles published in peer-reviewed journals; (II) studies providing data on GLS measured by STE in children or adolescents (excluding infants or children under 3 years of age) with overweight/obesity and preserved LVEF compared to healthy normal-weight individuals; (III) minimum set of clinical/demographic data.

Only studies enrolling normotensive children and adolescents with obesity were included. This restriction was applied to avoid the confounding influence of hypertension on LV mechanics, in order to specifically assess the effect of adiposity.

Two independent investigators, using the Newcastle–Ottawa Scale, assessed the methodological quality of each study (CC and EG). A Newcastle–Ottawa Scale score of seven or more was considered to suggest good quality.

### 2.2. Statistical Methods

Data were analyzed using Comprehensive Meta-Analysis software (v2.0). Continuous outcomes were pooled using standardized mean differences (SMD) with 95% confidence intervals (CI). Given the observed heterogeneity (I^2^ > 75%), a random-effects model (DerSimonian–Laird method) was applied. Sensitivity analyses were conducted by sequentially excluding individual studies to test robustness. Meta-regression analyses explored the relationship between GLS, LVEF, and clinical covariates (BMI, systolic BP, LV mass index). Publication bias was evaluated via funnel plot (Trim and Fill method) and Egger’s regression test.

As the present study focuses specifically on meta-regression results, funnel plots are not shown, but were reported in the primary analysis.

The review project, prospectively registered with the International Prospective Register of Systematic Reviews (PROSPERO CRD 42025635938), was performed according to the key recommendations provided by the Preferred Reporting Items of Systematic Reviews and Meta-Analyses (PRISMA) statement and Checklist 2020 [30]. The aim of the present sub-project, recorded by updating the main analysis PROSPERO identifier, was to investigate the potential factors associated with LV mechanics in childhood/adolescent obesity. For this purpose, meta-regression analysis was used to test the linear relationship between GLS and GCS with different indices of adiposity (i.e., BMI, fat mass, and waist circumference) and hemodynamic (i.e., systolic/diastolic BP and heart rate), metabolic (i.e., blood glucose, Homeostasis model assessment [HOMA], insulin, total cholesterol, low-density lipoprotein [LDL], and triglycerides), and echocardiographic variables (i.e., LVM index, mitral E/A and E/e’ ratio). Variables of interest were pre-specified and included in the analysis only if these were reported in at least four of the selected studies: this cutoff was chosen to minimize unstable variance estimates while retaining sufficient covariates for exploratory analyses [31]. Statistical significance was set at *p* < 0.05.

## 3. Results

**Figure 1:**
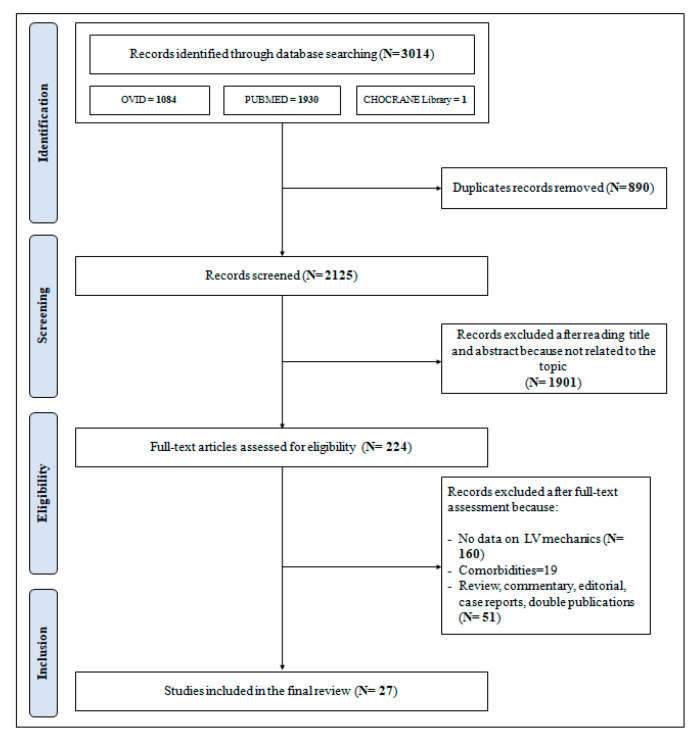
Preferred Reporting Items for Systematic reviews and Meta-Analyses (PRISMA) flow-chart for the selection of studies.

The PRISMA flowchart as presented in Figure 1 describes the full selection process that led to the inclusion of the studies selected for this analysis.

### 3.1. Population

A total of 1398 children/adolescents with overweight/obesity from 27 studies focusing on LV mechanics (i.e., GLS, GCS) were included in the analysis [32,33,34,35,36,37,38,39,40,41,42,43,44,45,46,47,48,49,50,51,52,53,54,55,56,57,58]. The Newcastle–Ottawa Score, used for assessing the quality of the studies, ranged from 7 to 9, the mean score being 7.3. Therefore, no studies were excluded based on their limited quality. A summary of the main results of the systematic review conducted for the primary analysis is provided in the Supplementary Materials [59].

Table 1 reports the main demographic/clinical and echocardiographic characteristics of the pooled population, highlighting the mean values of age, height, weight, BMI, office systolic/diastolic BP, LVEF, E/A, E/e’ ratio, GLS, and GCS. In particular, the mean value of LVEF and its lower–upper limit values were normal, demonstrating that all participants included in the selected studies had a well-preserved LV systolic function according to conventional echocardiography.

The prevalence of males in the pooled population was 53%. The mean age ranged from 4.0 to 17.8 years and the corresponding values of BMI and office systolic and diastolic BP ranged as follows: 23.7–36.7 Kg/m^2^, 97–128 mmHg, 57–78 mmHg.

Children with a history or clinical signs of cardiac disease, significant chronic illness, diabetes, renal disease, endocrinological disorders, systemic arterial hypertension, sleep apnea syndrome, familial hypercholesterolemia, and current use of cardiovascular medications were excluded from most studies (21 out of 27). Of note, the remaining six studies included subjects with obesity with and without comorbidities (i.e., diabetes, hypercholesterolemia, non-alcoholic fatty liver disease, cystic fibrosis, juvenile idiopathic arthrosis, and corrected coarctation of aorta). However, in the present analysis, sub-groups of children/adolescents with obesity with the above-mentioned comorbidities were excluded.

### 3.2. Meta-Regression Analysis

#### 3.2.1. LV Mechanics According to GLS Metric

**Figure 2:**
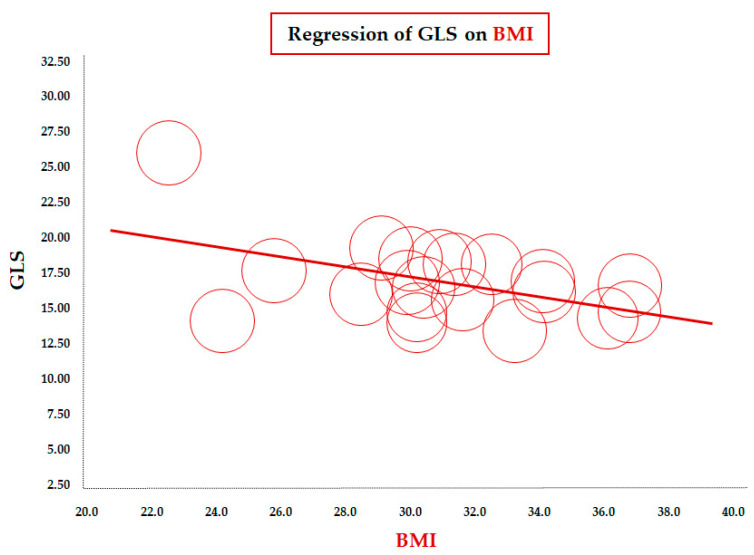
Meta-regression of global longitudinal strain (GLS) and body mass index (BMI) in children/adolescents > 10 years with obesity. Each circle represents one study.

Table 2 reports the results of the meta-regression between mean GLS values and demographic/clinical, laboratory, and echocardiographic variables. The meta-regression of GLS on BMI (data from 23 studies) showed a significant inverse correlation between the two parameters (coefficient = −0.33 ± 0.11, CI: −0.55/−0.11, *p* = 0.003). To assess this association in a more homogeneous population, we conducted a focused analysis in the subgroup of children older than 10 years. The inverse association remained significant in this subgroup of 20 studies (coefficient = −0.36 ± 0.14, 95% CI: −0.63/−0.08, *p* = 0.012), confirming the robustness of the relationship between increasing BMI and impaired longitudinal LV deformation during later childhood and adolescence (Figure 2).

**Figure 3:**
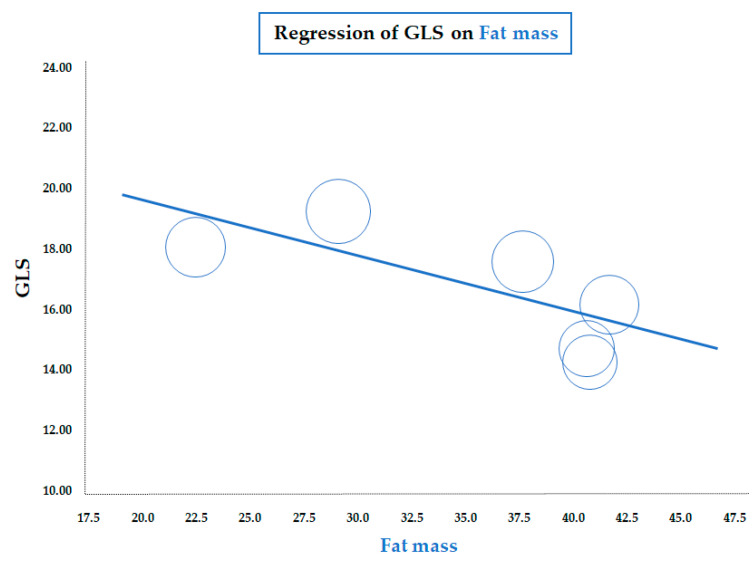
Meta-regression of global longitudinal strain (GLS) and body fat mass in children/adolescents with obesity. Each circle represents one study.

**Figure 4:**
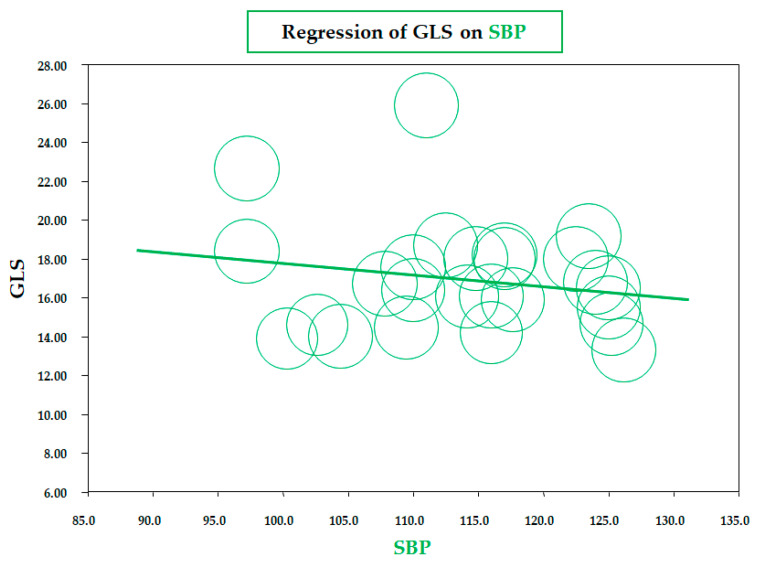
Meta-regression of global longitudinal strain (GLS) and systolic blood pressure (SBP) in children/adolescents with obesity. Each circle represents one study.

As for BMI, a similar significant correlation was observed for the meta-regression between GLS and body fat mass, assessed by a bioelectrical impedance analyzer or dual-energy X-ray absorptiometry (data from six studies; coefficient = −0.19 ± 0.07, CI: −0.32/−0.06, *p* = 0.005) (Figure 3). Therefore, both meta-regressions suggested that increasing adiposity indices corresponded to a reduction in LV longitudinal deformation, an expression of an early impairment of systolic function. On the contrary, the meta-regression of GLS and waist circumference (WC), height, heart rate, insulin, HOMA index, total cholesterol, LDL, triglycerides, LVMI, and E/A and E/e’ ratio did not reveal any significant association. Furthermore, in particular, no significant correlation was found between GLS and systolic BP (data from 25 studies, coefficient: −0.06 ± 0.06, CI: −0.18/0.06, *p* = 0.343) (Figure 4).

#### 3.2.2. LV Mechanics According to GCS Metric

The results of the meta-regression between the mean GCS values and several clinical, laboratory, and echocardiographic parameters are summarized in Table 3. Unlike the GLS, the meta-regression of GCS and BMI (data from 11 studies, coefficient = −0.26 ± 0.36, CI: −0.97/0.45, *p* = 0.470) and fat mass (data on 4 studies, coefficient = 0.14 ± 0.10, CI: −0.06/0.33, *p* = 0.161) did not show a significant association. This was also the case for the WC, fasting blood glucose, HOMA index, total cholesterol, LDL cholesterol, triglycerides, BP, heart rate, LVMI, and mitral E/A and E/e’ ratio.

## 4. Discussion

Myocardial strain imaging by STE is recognized as an accurate, sensitive, and reproducible tool for assessing cardiac function by measuring myocardial shortening in the longitudinal and circumferential directions and thickening in the radial direction [60]. Although multidirectional strains contribute to LV wall thickening, evaluating myocardial deformation for both clinical and research purposes remains mostly limited to measuring GLS only. This is because measurements of circumferential and radial strains from the short-axis views, despite improvements in the methodology, are less feasible and reproducible. In particular, it has been highlighted that the variability of the strain parameters is highest for the radial strain, followed by the circumferential and longitudinal strain [61]. Although studies targeting LV mechanics have consistently shown that this set of parameters provide higher clinical and prognostic information compared to LVEF in a broad array of clinical settings, findings regarding childhood obesity nowadays are scarce [62].

The present report, based on a large pooled population of normotensive children/adolescents with overweight/obesity without significant comorbidities enrolled in 27 studies since 2012, contributes to extending the current knowledge on factors associated with early alterations in LV mechanics in a clinical setting of increasing importance for the public health of future generations.

The meta-regression analyses revealed a significant inverse relationship between GLS and adiposity indexes such as BMI and fat mass. This means that the progressive increase in BMI in children/adolescents is associated with a proportional reduction in longitudinal myocardial deformation. The unfavorable effects of increased BMI on systolic function was further corroborated by the parallel inverse association between GLS and body fat mass assessed by bioelectrical impedance or X-ray absorptiometry. On the contrary, no correlation was found between GCS and these two adiposity indices. This result underscores the importance of a comprehensive multidirectional analysis of LV mechanics to collect important physio-pathological information on the natural history of early cardiac functional impairment in young normotensive subjects with obesity. In fact, it could be hypothesized that obesity-induced subclinical cardiac changes may affect myocytes differently in the three layers, affecting early on the endocardial layer responsible for longitudinal shortening, which is more vulnerable than the epicardial one assessed by circumferential shortening. This is in agreement with the concept that sub-endocardial longitudinal fibers are the most susceptible to humoral, endocrine, and hemodynamic alterations (i.e., increased preload and afterload) and ischemia, and their impairment may precede the reduction in mid-wall and epicardial myocardium function [63]. The mechanisms underlying the selective impairment of GLS in youth with obesity are likely multifactorial. Excess adiposity may lead to ectopic lipid accumulation in cardiomyocytes, promoting lipotoxicity and mitochondrial dysfunction [64]. In addition, systemic low-grade inflammation and insulin resistance have been shown to impair myocardial energetics and calcium handling. These processes may increase myocardial stiffness and impair longitudinal fiber shortening. Furthermore, early interstitial fibrosis has been documented in experimental models of obesity and may contribute to subclinical myocardial dysfunction, even in the absence of overt hypertension or structural remodeling [65].

Whether the lack of correlation between adiposity indices and GCS in children/adolescents actually reflects an involvement limited exclusively to the endocardial layer, the dysfunction of which is compensated for by the circumferential fibers, deserves to be explored further. Evidence regarding adult obesity conversely suggests a multidirectional involvement of myocardial deformation, likely reflecting the long-term impact of this condition on LV systolic function. A recent meta-analysis by our group revealed that longitudinal, mid-myocardial, and epicardial mechanics were significantly impaired in adult patients with obesity, highlighting that when obesity persists into adulthood, the cardiac changes uniformly affect all types of myocytes, irrespective of their direction and position in terms of myocardial layers [66].

It is worth noting that unlike BMI and fat mass, no correlation emerged between GLS and WC. Whether this finding supports the view that the WC metric less closely reflects the impact of obesity on LV mechanics in the pediatric setting remains purely hypothetical and, apparently, in contrast with the fact that WC has been shown to be more strongly correlated with visceral adipose tissue than BMI [67]. It should be emphasized, however, that WC cannot effectively distinguish between subcutaneous and visceral adipose tissue and the reliability of this measurement for assessing body fat in women is low.

A further novel observation of the present study comes from the meta-regression analyses, which have failed to reveal a significant relationship between LV mechanics and several metabolic variables such as blood glucose, insulin, HOMA index, cholesterol (total and LDL), and triglycerides. This supports the interpretation of a close, direct association between obesity and impaired LV mechanics completely independent of carbohydrate and lipid metabolism disorders and consistent with evidence that even children/adolescents with metabolically healthy obesity (MHO), the prevalence of which varies widely between 32 and 80%, may present with initial cardiac organ damage [68]. Genovesi et al. reported that children with MHO (defined according to an updated classification including uric acid and the HOMA index in addition to BP, blood glucose, HDL cholesterol, and triglycerides) had similar LVMI (i.e., 35.8 vs. 37.3 g/h^2.7^) and LVH prevalence (i.e., 35.6 vs. 36.3%) to their MUO counterparts [69].

Similarly to what emerged for metabolic variables, no association was found between GLS, GCS, and systolic/diastolic BP, thus excluding a synergistic adverse effect of office BP and obesity on LV mechanics. This finding is not surprising since the selected studies excluded participants with hypertension. Our rationale for restricting the analysis to normotensive children and adolescents with obesity was to isolate the independent effect of adiposity on LV mechanics, without the confounding impact of elevated BP. Accordingly, the BP values in the included cohorts were largely within the physiological range, which likely limited variability and precluded the detection of an association. Nevertheless, it should be remembered that it has been demonstrated that in healthy individuals, BP is also a key determinant of the variability of normal GLS values and increased afterload may affect myocardial function, leading to reduced LV longitudinal shortening [70]. Furthermore, regardless of the categorical definition of hypertension, it has often been observed that even “normotensive” children/adolescents with obesity have higher office and ambulatory BP values than normal-weight individuals [71].

Finally, the interpretation of a direct association between obesity and impaired LV mechanics is further supported by the findings provided by meta-regressions that did not find a significant relationship between GLS and LVMI or LV diastolic indexes. This would mean that the inverse relationship between obesity and GLS reflecting the unfavorable impact of the obese phenotype on LV systolic function is potentially independent of the morpho-functional alterations assessed with conventional echocardiography. The clinical implication of this observation is that the search for early alterations of myocardial deformation in the setting of pediatric obesity should be extended to subjects with normal cardiac structure and function phenotyped using LVMI, LVEF, or diastolic parameters. Therefore, GLS can be regarded as the key early marker for identifying initial LV systolic dysfunction in pediatric obesity and should be incorporated into echocardiographic evaluation to enhance early detection, screening, and risk stratification, regardless of concomitant risk factors such as hypertension or metabolic disturbances. In practice, abnormal GLS could (I) trigger intensified lifestyle interventions, (II) prioritize closer cardio-metabolic follow-up, and (III) serve as a quantitative intermediate endpoint to monitor the response to therapy.

An in-depth discussion targeting the mechanisms underlying pediatric obesity-related impaired myocardial deformation is beyond the aims of the present paper. Nonetheless, our findings allow us to make some interesting considerations of the impact of obesity on cardiac mechanics independently of comorbidities, alterations in glucose and lipid metabolism, and increased BP levels. Given that children/adolescents with obesity enrolled in the studies selected for this analysis can be defined as MHO, this raises the question of whether this phenotype is actually entirely devoid of adverse clinical implications. In the current literature, the relationship between obesity and excess cardiovascular risk is attributed to the interaction of multiple pathways such as endocrine, metabolic, inflammatory, and hemodynamic alterations. Regardless of evident metabolic, humoral, endocrine, and hemodynamic abnormalities resulting from the presence of comorbidities, it should be clearly emphasized that the increase in fat mass represents a metabolically active tissue leading to long-term cardiovascular damage.

Nowadays, adipose tissue is not considered just an energy depot and is now regarded as highly metabolically active; in particular, cardiac and abdominal adipose tissue acts as an endocrine organ by releasing myocardial growth and pro-inflammatory substances [72]. The expansion of adipose tissue as a consequence of excessive caloric intake is mediated by two processes characterized by adipocyte hyperplasia and hypertrophy. Both processes are associated with macrophage infiltration, reduced angiogenesis, tissue hypoxia, and a pro-inflammatory cytokine secretion pattern, with a correlation between adipose cell size and secretion of inflammatory mediators, such as tumor necrosis factor-⍺ (TNF-⍺), IL-6, and plasminogen activator inhibitor-1 [73]. Collectively, alterations in these pathways (which can exert their unfavorable effect even in an apparently normal metabolic context such as MHO) promote endoplasmic reticulum stress and mitochondrial dysfunction, resulting in a subtle decline in myocardial contractility, only detectable by assessing LV mechanics.

From a clinical perspective, these findings highlight the potential application of myocardial strain assessment as part of cardiovascular screening in children and adolescents with obesity, enabling detection of subclinical dysfunction earlier than is possible with conventional echocardiography. Furthermore, GLS could represent a useful surrogate endpoint in interventional studies, providing a sensitive marker to monitor the effects of lifestyle modifications or emerging pharmacological strategies in this high-risk population.

### Limitations of the Study

Our report is faced with several limitations. We did not analyze individual data from the original databases but only the results derived from revised papers, which may introduce ecological bias and limit the strength of causal inference. Second, all included studies were cross-sectional in design, so temporal and causal relationships between obesity and alterations in LV mechanics cannot be definitively established.

The associations derived from the meta-regressions are observational, and have a weaker interpretation than the causal relationships derived from randomized comparisons. This applies particularly when averages of patient characteristics in each trial are used as covariates in the regression. In particular, the association between GLS and fat mass should be interpreted cautiously, as only six studies provided usable data for this meta-regression, potentially limiting the statistical power and robustness of the conclusion.

Another limitation was the heterogeneity across studies in terms of population characteristics, definitions of overweight/obesity, echocardiographic equipment, and strain analysis protocols, which may have influenced the pooled estimates.

In particular, a limitation of our analysis is the use of BMI expressed in kg/m^2^ rather than BMI-for-age z-scores, which are generally preferred in pediatric populations because they account for age-, sex-, and growth-related changes in body composition, but most of the included studies did not report BMI z-scores. While BMI in kg/m^2^ remains widely used in clinical practice and retains prognostic value, it may misclassify adiposity during periods of rapid growth, particularly in puberty. Future research should prioritize BMI z-scores or other age- and sex-adjusted indices to more accurately assess adiposity and its relationship with cardiac mechanics in children and adolescents.

Finally, the restriction to papers published in English due to difficulties in retrieving and interpreting papers in other languages may have partly affected our findings.

## 5. Conclusions

In summary, our meta-regression demonstrates that adiposity parameters are associated with reduced LV longitudinal deformation in children and adolescents with obesity, whereas other clinical and echocardiographic parameters were not. These findings underline the potential role of GLS as an early marker of subclinical myocardial dysfunction in pediatric obesity. Clinically, incorporating GLS into routine echocardiographic evaluations may improve early detection, screening, and risk stratification in this high-risk group, guiding timely preventive or therapeutic interventions. Future longitudinal studies are warranted to establish the prognostic value of GLS alterations, and interventional trials should determine whether lifestyle modification or pharmacological strategies can reverse these early myocardial changes.

## Figures and Tables

**Table 1:** Clinical, laboratory, and echocardiographic characteristics of the pooled overweight/obese pediatric population.

Variables	N Studies	N Subjects	Mean Values ± SE	Lower–Upper Limit
Age (years)	27	1398	12.6 ± 1.8	9.0/16.2
Height (cm)	16	891	150.3 ± 6.7	137.1/163.6
Weight (kg)	15	863	67.3 ± 7.8	51.8/82.8
BMI (kg/m^2^)	23	1214	29.9 ± 1.3	27.4/32.4
SBP (mmHg)	25	1294	113.8 ± 2.3	109.3/118.4
DBP (mmHg)	24	1235	67.5 ± 1.3	64.9/70.1
LVEF (%)	22	1159	64.5 ± 0.7	63.2/65.8
LVMI (g/h^2.7^)	15	709	35.3 ± 6.4	22.7/47.8
LVMI (g/m^2^)	10	605	78.7 ± 4.9	69.0/88.3
E/A ratio	19	1092	1.79 ± 0.06	1.67/1.91
E/e’ ratio	13	739	7.04 ± 0.38	6.29/7.79
GLS (%)	27	1398	17.1 ± 0.5	16.1/18.1
GCS (%)	13	672	19.0 ± 0.1	17.1/20.9

BMI = body mass index; DBP = diastolic blood pressure; SBP = systolic blood pressure; LVEF = left ventricular ejection fraction; LVMI = left ventricular mass index; E/A = early to late mitral flow ratio; E/e’ = early mitral flow to annular mitral velocity ratio; GLS = global longitudinal strain; GCS = global circumferential strain.

**Table 2:** Meta-regression of global longitudinal strain (GLS) on clinical, laboratory, and echocardiographic variables in obese children/adolescents.

Meta-Regression	N Studies	N Subjects	Coefficient	Lower–Upper Limits	*p* Value
BMI (kg/m^2^)	23	1214	−0.329 ± 0.111	−0.547/−0.111	0.003
Fat Mass (%)	6	222	−0.187 ± 0.067	−0.318/−0.056	0.005
WC (cm)	8	498	−0.044 ± 0.068	−0.178/0.089	0.516
Height (cm)	16	891	−0.068 ± 0.051	−0.167/0.032	0.183
SBP (mmHg)	25	1294	−0.060 ± 0.063	−0.183/0.063	0.343
DBP (mmHg)	24	1235	−0.128 ± 0.098	−0.320/0.064	0.192
HR (b/min)	19	896	−0.022 ± 0.081	−0.181/0.137	0.792
Blood glucose (mg/dL)	14	692	−0.043 ± 0.045	−0.131/0.046	0.345
Insulin (mU/L)	13	497	0.299 ± 0.161	−0.016/0.14	0.063
HOMA IR	11	480	0.274 ± 0.774	−1.242/1.790	0.723
Total Cholesterol (mg/dL, mml/L)	13	851	0.022 ± 0.027	−0.031/0.075	0.408
LDL Cholesterol (mg/dL, mml/L)	11	771	0.024 ± 0034	−0.043/0.091	0.476
Triglycerides (mg/dL, mml/L)	10	816	−0.015 ± 0.025	−0.064/0.034	0.550
LVMI (g/h^2.7^)	15	709	−0.018 ± 0.050	−0.116/0.079	0.711
E/A ratio	19	1092	1.855 ± 2.338	−2.726/6.437	0.427
E/e’ ratio	13	800	−0.014 ± 0.500	−0.993/0.965	0.977

BMI = body mass index; SBP = systolic blood pressure; DBP = diastolic blood pressure; HR = heart rate; WC = waist circumference; HOMA = homeostatic model assessment; LDL = low-density lipoprotein; LVMI = left ventricular mass index; E/A = early diastolic peak flow mitral velocity, late diastolic peak flow mitral velocity; E/e’ = mitral flow velocity to annular velocity.

**Table 3:** Meta-regression of global circumferential strain (GCS) on clinical, laboratory, and echocardiographic variables in obese children/adolescents.

Meta-Regression	N Studies	N Subjects	Coefficient	Lower–Upper Limits	*p* Value
BMI (kg/m^2^)	11	567	−0.261 ± 0.361	−0.969/0.447	0.470
Fat Mass (%)	4	141	0.135 ± 0.098	−0.057/0.326	0.169
Height (cm)	10	527	−0.103 ± 0.103	−0.306/0.099	0.317
SBP (mmHg)	11	548	−0.118 ± 0.161	−0.433/0.197	0.464
DBP (mmHg)	11	548	0.190 ± 0.216	−0.234/0.614	0.379
HR (b/min)	7	418	−0.020 ± 0.187	−0.386/0.346	0.914
WC (cm)	6	402	−0.036 ± 0.209	−0.445/0.374	0.862
Blood Glucose (mg/dL)	8	477	−0.347 ± 0.232	−0.801/0.107	0.134
Insulin (mU/L)	6	253	−0.072 ± 0.178	−0.421/0.278	0.688
HOMA IR	6	253	−0.665 ± 0.759	−2.154/0.822	0.381
Total Cholesterol (mg/dL, mml/L)	9	477	0.086 ± 0.040	0.009/0.164	0.9
LDL Cholesterol (mg/dL, mml/L)	8	437	0.084 ± 0.041	0.004/0.165	0.9
Triglycerides (mg/dL, mml/L)	8	442	−0.073 ± 0.112	−0.292/0.145	0.511
LVMI (g/h^2.7^)	7	299	0.028 ± 0.062	−0.093/0.149	0.647
E/A ratio	11	612	−1.984/4.142	−10.102/6.134	0.632
E/e’ ratio	9	539	0.233 ± 0.792	−1.320/1.785	0.769

BMI = body mass index; SBP = systolic blood pressure; DBP = diastolic blood pressure; HR = heart rate; WC = waist circumference; HOMA = homeostatic model assessment; LDL = low-density lipoprotein; LVMI = left ventricular mass index; E/A = early diastolic peak flow mitral velocity, late diastolic peak flow mitral velocity; E/e’ = mitral flow velocity to annular velocity.

## Data Availability

Available upon request to the corresponding author. The review protocol is available at https://www.crd.york.ac.uk/PROSPERO/view/CRD42025635938 (accessed on 2 April 2025), while the review data can be provided upon reasonable request.

## Supplementary Materials

The following supporting information can be downloaded at: https://www.mdpi.com/article/10.3390/diseases13090301/s1, File S1: Key Results of the Primary Analysis.
