# Supplementary material for: Targeting Myocardial Mechanics in Children and Adolescents with Obesity and Non-Elevated Blood Pressure: A Meta-Regression Study
Source: Diseases. 2025 Sep 11;13(9):301. doi: 10.3390/diseases13090301 (PMC12468501; doi:10.3390/diseases13090301)
Supplement: Supplementary file 1 [file diseases-13-00301-s001.zip › diseases-3801930-supplementary.pdf]

Supplementary File

Key Results of the Primary Analysis, available at https://doi.org/10.1007/s40292-025-00726-9

>Main results:

- GLS was significantly lower in individuals with overweight/obesity compared to controls (SMD: –1.28±0.14; 95% CI: –1.57 to –1.00; $p < 0.001$), while LVEF also showed a small but statistically significant reduction (SMD: –0.14±0.04; $p < 0.001$).
- Individuals with overweight/obesity also showed worse diastolic function, with a significantly reduced E/A ratio (SMD: –0.25±0.04) and increased E/e′ (SMD: 0.50±0.16).
- LV mass indexed to $height^{2.7}$ was significantly higher in the obese group (SMD: 1.10±0.14; $p < 0.001$).
- To assess the effect of individual studies on the pooled results, we conducted a sensitivity analysis by sequentially excluding each study and recalculating the combined estimates. This procedure did not alter the pooled estimates.
- The meta-regression revealed a significant inverse correlation between GLS and BMI (β = –0.33±0.11; $p = 0.003$), while no significant associations were found between LVEF and BMI, or between GLS and systolic BP or LVMI.
